# Influence of emotional regulation capacity on loneliness among university students: the suppressing effect of solitude coping and the mediating effect of solitude comfort

**DOI:** 10.3389/fpsyg.2026.1842308

**Published:** 2026-07-09

**Authors:** Zanru Yang, Chunguang Liang, Dandan Wang, Suyan Wang

**Affiliations:** 1 The First Affiliated Hospital of Jinzhou Medical University, Jinzhou Liaoning, China; 2 Department of Life and Health, Huzhou College, Huzhou, Zhejiang, China; 3 Graduate School of Jinzhou Medical University, Jinzhou, Liaoning, China; 4 Mental Health Guidance Center, Jinzhou Medical University, Jinzhou, Liaoning, China

**Keywords:** college students, emotional regulation capacity, loneliness, solitude comfort, solitude coping

## Abstract

**Background:**

Contemporary college students often experience significant loneliness. This study was aimed to explore the role of solitude capacity in the association between emotional regulation capacity and loneliness of college students.

**Methods:**

From June 10th to July 5th 2025, 814 college students were selected to participate in a questionnaire survey using Emotion Regulation Scale (ERSQ), the solitude capacity scale (SCS), and the UCLA Loneliness Scale. The suppressing effect and the mediating effects of solitude coping and solitude comfort between college students' emotional regulation capacity and loneliness were tested.

**Results:**

The score of the emotional regulation capacity of university students was (69.65 ± 16.56), the score of solitude capacity was (55.92 ± 7.06), the score of solitude coping was (27.39 ± 4.04), the score of solitude comfort was (28.52 ± 3.87), and the score of loneliness was (40.43 ± 10.82). Emotional regulation capacity was significantly positively correlated with solitude and its two dimensions (*P* < 0.01), and significantly negatively correlated with loneliness (*r* = −0.269, *P* < 0.01). Solitude capacity and loneliness were not significantly correlated (*r* = 0.057, *P*> 0.05). However, solitude coping was significantly positively correlated with loneliness (*r* = 0.186, *P* < 0.01), and solitude comfort was significantly negatively correlated with loneliness (*r* = −0.090, *P* < 0.01). Solitude coping showed a suppressing effect between emotional regulation capacity and loneliness, with the suppressing effect accounting for 22.54% of the direct effect, while solitude comfort showed a mediating effect between emotional regulation capacity and loneliness, with the mediating effect accounting for 20.81% of the direct effect.

**Conclusions:**

Solitude coping suppresses the relationship between emotional regulation capacity and loneliness, while solitude comfort mediates this relationship.

## 1 Introduction

University students are at a critical stage of personality development, where establishing intimate relationships and cultivating positive interpersonal connections are core developmental tasks for this phase. However, an increasing number of studies show that contemporary college students often experience significant loneliness (Zhu et al., 2023; Twenge et al., 2021). Loneliness refers to the unpleasant emotional experience individuals feel when they perceive deficiencies in either the quantity or quality of their social network (Krieger, 2024). Research indicates that loneliness is closely associated with various adverse outcomes, such as depression, anxiety, impaired cognitive function, and decreased subjective wellbeing (Lawrence, 2024). Studies further reveal that the detrimental effects of loneliness on individual mental health are long-term and profound, potentially even exerting lifelong impacts on an individual's mental health level (Mann et al., 2022). Therefore, loneliness has become a key indicator for assessing the mental health of university students. Exploring the mechanisms underlying loneliness holds significant theoretical and practical importance for safeguarding the mental health of this population.

Emotion regulation refers to an individual's ability to effectively cope with psychological stress using cognitive and behavioral strategies (Wang and Chu, 2022). Effective emotion regulation can significantly improve negative emotional experiences and behavioral expressions, serving as an important psychological resource for university students (Demichelis et al., 2024; Musella et al., 2025). Enhancing individuals' emotional regulation capacity is of great significance in alleviating various negative emotional experiences (Wang et al., 2024), including loneliness (Patrichi et al., 2025). The level of emotional regulation capacity is of great significance to an individual's social adaptation. High emotional regulation capacity helps to promote post-traumatic growth in students (Kim et al., 2023). Emotional regulation capacity deficiency may lead to various psychological and emotional disorders, such as post-traumatic stress disorder (Kim et al., 2023), negative emotions (Wang and Chu, 2022), and depression (Peng and Ishak, 2024). Regarding the relationship between emotional regulation capacity and loneliness, there may be a bidirectional association between the two. On one hand, studies have shown that individuals with high levels of loneliness tend to employ maladaptive emotion regulation strategies (Korkmaz et al., 2025), on the other hand, the use of ineffective emotion regulation strategies, such as emotional suppression or social avoidance, can increase the difficulty for individuals in establishing social connections, thereby significantly exacerbating feelings of loneliness (Patrichi et al., 2025). Based on the above reasoning, this study proposes the following hypothesis:

**Hypothesis 1:** Emotional regulation capacity has a significant negative correlation with loneliness among university students.

Solitude capacity refers to an individual's capacity to be alone and experience a sense of openness, comfort, and ease during solitude (Larson and Lee, 1996). Generally, solitude capacity can be divided into two dimensions: solitude coping and solitude comfort (Larson and Lee, 1996; Lin et al., 2020). The coping dimension assesses how individuals use solitude to handle stress, while the comfort dimension evaluates whether individuals feel at ease when alone (Larson and Lee, 1996). Solitude coping and solitude comfort may have different implications for loneliness. Solitude comfort captures the extent to which individuals feel relaxed, open, and at ease when alone, whereas solitude coping reflects the tendency to use solitude as a way to manage stress or negative emotions (Larson and Lee, 1996; Lin et al., 2020). Therefore, solitude coping may not necessarily represent intrinsic enjoyment of solitude; rather, it may reflect a more instrumental and stress-oriented coping strategy. From the perspective of emotion regulation and emotional invalidation, distress is regulated not only through internal cognitive and behavioral strategies, but also through emotional expression, validation, and interpersonal support (Rezaei, 2025; Rezaei et al., 2024, 2025). When individuals rely heavily on being alone to cope with distress, they may temporarily reduce emotional overload, but they may also have fewer opportunities to disclose negative emotions, receive emotional validation, and obtain social support from others. In this sense, solitude coping may be associated with higher loneliness, particularly when it functions as a substitute for interpersonal connection rather than as a restorative form of solitude. Thus, although emotional regulation capacity may be positively associated with solitude coping, solitude coping may in turn be positively associated with loneliness. This opposite-direction pathway may offset part of the negative association between emotional regulation capacity and loneliness, which is consistent with a suppression effect, also known as inconsistent mediation (MacKinnon et al., 2000). According to Self-Determination Theory, individuals with strong emotional regulation capacity possess higher self-esteem and self-identity (Fernandes et al., 2022), and good self-identity is conducive to the development of solitude capacity (Lian et al., 2021; van den Brink et al., 2020). Notably, while some studies suggest that high solitude capacity can alleviate negative psychological processes (Palgi et al., 2021), others confirm that solitude capacity may be a double-edged sword: the comfort dimension can relieve stress generated by intimate relationships, whereas the coping dimension may enhance loneliness through social avoidance (Lian et al., 2021), reflecting the complexity of the impact of solitude capacity on individual mental health (Lin et al., 2020). Although a substantial body of research has examined loneliness and solitude, several gaps remain. First, previous studies have mainly focused on the prevalence, adverse consequences, and interpersonal correlates of loneliness (Hawkley, 2010; Zahedi et al., 2022; Calderon Leon et al., 2024), whereas less attention has been paid to how individual psychological resources, such as emotional regulation capacity, are associated with loneliness (Patrichi et al., 2025). Second, solitude has often been treated as either beneficial or harmful, but fewer studies have distinguished between different dimensions of solitude capacity (Lin et al., 2020; Weinstein and Hansen, 2023). In particular, solitude coping and solitude comfort may have opposite implications for loneliness (Lin et al., 2020). Third, limited evidence has examined whether these two dimensions operate simultaneously in the association between emotional regulation capacity and loneliness. Therefore, by distinguishing solitude coping from solitude comfort and testing their opposing indirect pathways, the present study may add to the literature by clarifying the differentiated role of solitude capacity in the loneliness experience of university students. Based on the above rationales, this study proposes the following hypotheses:

**Hypothesis 2:** The solitude comfort dimension of solitude capacity may mediate the effect of emotional regulation capacity on loneliness, i.e., a mediating effect.

**Hypothesis 3:** The solitude coping dimension of solitude capacity may play an obstructive role, i.e., a suppressing effect.

**Figure 1:**
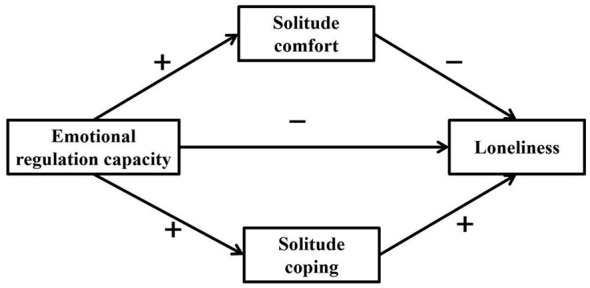
Diagram for proposed research hypothesis.

In summary, this study aims to explore the internal mechanism through which emotion regulation capacity is associated with loneliness among university students, focusing on analyzing the mediating effect of the solitude comfort dimension and the suppressing effect of the solitude coping dimension of solitude capacity (Figure 1). Through these analyses, this study intends to provide references and bases for improving university students' experience of loneliness, enhancing their mental health levels, and preventing psychological crises on campus.

## 2 Methods

### 2.1 Participants and procedure

Full-time undergraduate students from one public university were selected as the research subjects. The survey was conducted based on a convenient sampling strategy using Qustionnaire Star (Wenjuanxing in Chinese, wjx.cn) for questionnaire design, and relied on social tools such as WeChat and QQ for distribution from June 10th to July 5th, 2025. Inclusion criteria: (1) enrolled university students; (2) aged 18 or above; (3) with normal intellectual and clear thinking abilities. Exclusion criterion: suffering from severe mental illness. This study complied with the Declaration of Helsinki and was reviewed and approved by the Ethics Committee of Jinzhou Medical University (No. JZMULL2025226). The informed consent was obtained from all participants.

## 3 Measures

### 3.1 General information questionnaire

A self-designed questionnaire was used to collect information including age, gender, ethnicity, grade, place of origin, only-child status, and parental marital status.

### 3.2 Emotion regulation skill questionnaire (ERSQ)

Developed by Berking and Znoj (2008)) this scale consists of 27 items scored on a 5-point Likert scale ranging from “0 = never” to “4 = almost always.” The Chinese version of this measure had good psychometric performance among university students (Ma et al., 2025). In this study, the Cronbach's α coefficient of the scale was 0.960.

### 3.3 University of California at Los Angeles loneliness scale (UCLA)

Developed by (Russell and Loneliness Scale 1996), this scale was tested and revised by Liu Tianyin et al. in the Chinese cultural context (Liu et al., 2020). It consists of 20 items, with 9 reverse-scored items. It uses a 4-point Likert scale ranging from “1 = never” to “4 = always,” with a total score of 80. Higher scores indicate greater loneliness. In this study, the Cronbach's α coefficient of the scale was 0.931.

### 3.4 Solitude capacity scale (SCS)

Developed by Larson and Lee (1996) and revised by Wu Lijuan in Chinese cultural context (Lin et al., 2020), this scale consists of 20 items, including 5 reverse-scored items. It comprises two subscales: Solitude Coping and Solitude Comfort. The first 10 items constitute the Solitude Coping subscale, focusing on how individuals use solitude to handle stress (e.g., “Staying away from people helps me contemplate current problems”). Items 11–20 form the Solitude Comfort subscale, assessing personal comfort during solitude (e.g., “When I am alone, I am satisfied with myself”). The scale uses a 4-point Likert scale ranging from “1 = strongly disagree” to “4 = strongly agree,” with a total score of 80. Higher scores indicate stronger solitude capacity. In this study, the Cronbach's α coefficient of the scale was 0.848.

### 3.5 Statistical analysis

SPSS 25.0 was used for data management and analysis. Descriptive statistical analysis was conducted for the variables involved in the study. Normally distributed continuous data were expressed as mean ± standard deviation, while categorical data were expressed as frequencies (%). Harman's single-factor test was used before data analysis to assess common method bias (Podsakoff et al., 2003). The results showed 10 factors with eigenvalues greater than 1, with the largest factor explaining 23.57% of the variance, well below the 40% critical threshold, indicating no significant common method bias in this study. Independent samples *t*-tests and one-way ANOVA were used for difference testing. Pearson correlation analysis was performed. Hayes' PROCESS macro was employed to conduct suppression effect and mediation effect analyses using the Bootstrap method. The Bootstrap resampling was set to 5,000 repetitions, with a 95% confidence interval. A *P*-value <0.05 was considered statistically significant.

## 4 Results

### 4.1 Demographic characteristics of university students and their scores on emotional regulation capacity solitude capacity, and loneliness

A total of 814 participants were included for final analysis. Among them, 606 were female (74.5%). Ages ranged from 18 to 25 years, with a mean age of 19.97 years and a median of 20 years. There were 664 Han Chinese participants, accounting for 81.6%. By grade: 214 were 1st-year students (26.3%), 278 were 2nd-year students (34.2%), 151 were 3rd-year students (18.6%), 155 were 4th-year students (19.0%), and 16 were in their 50th year (2.0%). Regarding place of origin, 511 were from rural areas, accounting for 62.8%. There were 309 only-children, accounting for 38.0%. Participants from divorced families numbered 83 (10.2%), while those from non-divorced families numbered 731 (89.8%).

The mean score for college students' emotion regulation ability was 69.65 ± 16.56. The mean score for solitude capacity was 55.92 ± 7.06, with subscale scores of 27.39 ± 4.04 for Solitude Coping and 28.52 ± 3.87 for Solitude Comfort. The mean score for loneliness was 40.43 ± 10.82.

Male students had higher emotional regulation capacity than females (*P* = 0.023). One-way ANOVA revealed significant differences across grade levels in the variables of emotional regulation capacity, solitude capacity, solitude coping, solitude comfort, and loneliness (all *Ps* < 0.01). *Post-hoc* tests using Tukey HSD revealed that the emotion regulation capacity of sophomores, juniors, and seniors were significantly better than those of freshmen (all *Ps* < 0.01); the solitude capacity of juniors and seniors is significantly better than that of freshmen (all *Ps* < 0.01); the solitude coping of juniors and seniors is significantly better than that of freshmen (all *Ps* < 0.05); the solitude comfort for junior, senior, and fifth-year students is significantly better than that of freshmen (all *Ps* < 0.01); the loneliness of sophomore, junior, and senior students have significantly lower than freshmen (all *Ps* < 0.05). Students from urban areas had higher emotional regulation capacity, solitude capacity, solitude coping, and solitude comfort than those from rural areas (all *Ps* < 0.01). Only children had higher emotional regulation capacity, solitude capacity, solitude coping, and solitude comfort than non-only children (all *Ps* < 0.05). Students from divorced families had higher perception of loneliness than those from not-divorced families (*P* = 0.004). Detailed information are shown in Table 1.

**Table 1:** Analysis of differences in emotional regulation capacity, solitude capacity and loneliness among different demographic characteristics (*n* = 814).

Variables	Category	*n*	Statistical values	Emotional regulation capacity	Solitude ability	Solitude coping	Solitude comfort	Loneliness
**Gender**	**Male**	208	M ± SD	71.89 ± 17.55	56.64 ± 7.65	27.87 ± 4.49	28.78 ± 4.15	40.62 ± 11.66
	**Female**	606	M ± SD	68.88 ± 16.16	55.67 ± 6.83	27.23 ± 3.87	28.43 ± 3.77	40.37 ± 10.53
			t	**2.27**	1.73	1.96	1.11	0.28
			p	**0.023**	0.084	0.051	0.268	0.776
**Ethnicity**	**Han**	664	M ± SD	69.31 ± 16.41	56.07 ± 6.90	27.52 ± 3.98	28.56 ± 3.83	40.55 ± 10.86
	**Ethnic minority**	150	M ± SD	71.13 ± 17.19	55.23 ± 7.71	26.85 ± 4.26	28.37 ± 4.07	39.89 ± 10.68
			*t*	−1.22	1.32	1.81	0.52	0.68
			*p*	0.224	0.186	0.07	0.603	0.495
**Grade (school year)**	**First**	214	M ± SD	63.66 ± 16.78	54.33 ± 7.09	26.74 ± 4.12	27.58 ± 3.82	43.52 ± 9.73
	**Second**	278	M ± SD	71.64 ± 16.09	55.56 ± 6.93	27.15 ± 3.83	28.41 ± 3.87	39.29 ± 10.73
	**Third**	151	M ± SD	71.68 ± 15.03	57.16 ± 6.81	28.24 ± 3.97	28.92 ± 3.46	40.37 ± 10.67
	**Fourth**	155	M ± SD	72.00 ± 17.13	57.27 ± 6.96	27.91 ± 3.95	29.36 ± 3.95	38.40 ± 11.52
	**Fifth**	16	M ± SD	73.19 ± 11.15	58.38 ± 7.65	27.25 ± 6.22	31.13 ± 4.40	39.13 ± 13.26
			*F*	**9.95**	**6.12**	**3.97**	**7.46**	**6.875**
			*p*	**<0.001**	**<0.001**	**0.003**	**<0.001**	**<0.001**
**Place of origin**	**Rural**	511	M ± SD	68.36 ± 15.94	55.19 ± 6.72	27.04 ± 3.89	28.15 ± 3.67	40.67 ± 10.34
	**Urban**	303	M ± SD	71.82 ± 17.38	57.14 ± 7.45	27.99 ± 4.23	29.15 ± 4.12	40.03 ± 11.60
			*t*	**−2.9**	**−3.84**	**−3.26**	**−3.58**	0.82
			*p*	**0.004**	**<0.001**	**0.001**	**<0.001**	0.415
**Only child**	**Yes**	309	M ± SD	71.43 ± 17.59	56.91 ± 7.41	27.92 ± 4.30	28.99 ± 4.01	39.79 ± 11.62
	**No**	505	M ± SD	68.56 ± 15.82	55.30 ± 6.77	27.07 ± 3.84	28.23 ± 3.76	40.83 ± 10.30
			*t*	**2.41**	**3.17**	**2.92**	**2.73**	−1.33
			*p*	**0.016**	**0.002**	**0.004**	**0.007**	0.184
**Parental marital status**	**Divorced**	83	M ± SD	71.40 ± 16.60	55.67 ± 7.77	27.34 ± 4.58	28.34 ± 4.05	43.64 ± 10.86
	**Not divorced**	731	M ± SD	69.45 ± 16.56	55.94 ± 6.98	27.40 ± 3.98	28.54 ± 3.85	40.07 ± 10.77
			*t*	1.02	−0.33	−0.13	−0.46	**2.86**
			*p*	0.31	0.743	0.895	0.647	**0.004**

Bold values indicate statistically significant differences at the α = 0.05 level.

### 4.2 Correlations among emotion regulation ability, solitude capacity, and loneliness

The results of correlation analysis among variables showed that: College students' emotional regulation capacity was positively correlated with solitude capacity (*r* = 0.254), solitude coping (*r* = 0.178) and solitude comfort (*r* = 0.278) (all *Ps* < 0.01), and negatively with loneliness (*r* = −0.269, *P* < 0.01). The correlation between solitude capacity and loneliness was not statistically significant (*r* = 0.057, *P* > 0.05). However, solitude coping was positively correlated with loneliness (*r* = 0.186, *P* < 0.01), while solitude comfort was negatively correlated with loneliness (*r* = −0.090, *P* < 0.01) (Table 2). The non-significant association between overall solitude capacity and loneliness may be explained by the opposite directions of its two dimensions. Specifically, solitude coping was positively associated with loneliness, whereas solitude comfort was negatively associated with loneliness. When these two dimensions were combined into the total solitude capacity score, their opposing associations may have offset each other, resulting in a non-significant total-score association.

**Table 2:** Correlation between emotional regulation capacity, solitude capacity, solitude comfort, solitude coping and loneliness (*n* = 814).

Variables	1	2	3	4	5
**1 Emotional regulation capacity**	1				
**2 Solitude capacity**	0.254^**^	1			
**3 Solitude coping**	0.178^**^	0.897^**^	1		
**4 Solitude comfort**	0.278^**^	0.887^**^	0.591^**^	1	
**5 Loneliness**	−0.269^**^	0.057	0.186^**^	−0.090^**^	1

^**^P < 0.01.

### 4.3 The suppressing effect of solitude coping and the mediating effect of solitude comfort in the relationship between emotional regulation capacity and loneliness among college students

Based on the comparative results of differences in this study, Model 4 of the PROCESS macro in SPSS 25.0 was used to test the suppressing effect and mediating effect. The results are shown in Table 3. Regression analysis was conducted with loneliness as the dependent variable and emotional regulation capacity as the independent variable, revealing a significant regression equation with a regression coefficient of −0.170 (*P* < 0.001). Regression analysis with solitude coping as the dependent variable and emotional regulation capacity as the independent variable showed a significant regression equation with a regression coefficient of 0.039 (*P* < 0.001). Similarly, regression analysis with solitude comfort as the dependent variable and emotional regulation capacity as the independent variable demonstrated a significant regression equation with a regression coefficient of 0.059 (*P* < 0.001). Furthermore, regression analysis with loneliness as the dependent variable and solitude coping, solitude comfort, and emotional regulation capacity as independent variables yielded a significant regression equation, with regression coefficients of 0.998 (*P* < 0.001), −0.606 (*P* < 0.001), and −0.173(*P* < 0.001), respectively.

**Table 3:** Regression analysis of the relationships among variables (*n* = 814).

Regression equation		Overall fit index			Significance of regression coefficient		
Outcome variable	Predictive variable	*R^2^*	*F*	*P*	*B*	*t*	*P*
**Loneliness**	**Constant**	0.096	12.237	<0.001	63.329	15.44	<0.001
	**Emotional regulation capacity**				−0.170	−7.63	<0.001
	**Gender**				−1.356	−1.60	0.110
	**Ethnicity**				−0.247	−0.26	0.793
	**Grade**				−0.967	−2.82	0.005
	**Place of origin**				0.496	0.59	0.557
	**Only child**				0.466	0.55	0.582
	**Parental marital status**				−3.967	−3.29	0.001
**Solitude coping**	**Constant**	0.054	6.507	<0.001	25.381	16.19	<0.001
	**Emotional regulation capacity**				0.039	4.56	<0.001
	**Gender**				−0.322	−0.99	0.320
	**Ethnicity**				−0.725	−2.02	0.044
	**Grade**				0.209	1.60	0.111
	**Place of origin**				0.481	1.49	0.136
	**Only child**				−0.412	−1.28	0.202
	**Parental marital status**				0.126	0.27	0.785
**Solitude comfort**	**Constant**	0.103	13.204	<0.001	22.601	15.46	<0.001
	**Emotional regulation capacity**				0.059	7.42	<0.001
	**Gender**				0.082	0.27	0.786
	**Ethnicity**				−0.294	−0.88	0.380
	**Grade**				0.426	3.48	0.001
	**Place of origin**				0.494	1.64	0.101
	**Only child**				−0.166	−0.55	0.583
	**Parental marital status**				0.318	0.74	0.459
**Loneliness**	**Constant**	0.186	20.339	<0.001	51.699	11.22	<0.001
	**Solitude coping**				0.998	9.439	<0.001
	**Solitude comfort**				−0.606	−5.32	<0.001
	**Emotional regulation capacity**				−0.173	−7.91	<0.001
	**Gender**				−0.985	−1.22	0.222
	**Ethnicity**				0.299	0.33	0.739
	**Grade**				−0.918	−2.79	0.005
	**Place of origin**				0.315	0.39	0.605
	**Only child**				0.777	0.97	0.334
	**Parental marital status**				−0.390	−3.40	<0.001

**Figure 2:**
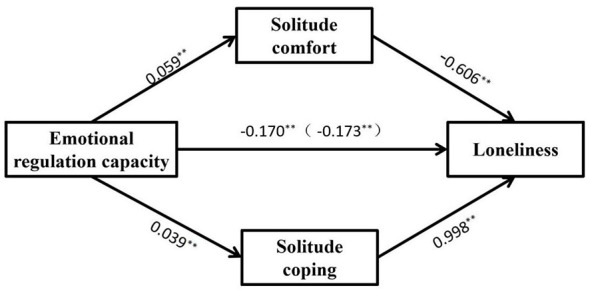
Result of the mediation analyses. **P < 0.01.

Based on correlation and regression analyses, the suppressing and mediating effects were examined using PROCESS macro Model 4, with emotional regulation capacity as the independent variable, solitude coping and solitude comfort as mediators, and loneliness as the dependent variable. The results, presented in Table 4 and Figure 2, indicated that solitude coping exhibited a suppressing effect, while solitude comfort demonstrated a mediating effect. The total effect was −0.170, and the direct effect was −0.173. The indirect effect through solitude coping was positive (0.039), whereas the indirect effect through solitude comfort was negative (−0.036). These two pathways nearly offset each other, resulting in a very small total indirect effect (0.003).

**Table 4:** Mediating model test for loneliness(*n* = 814).

Effect type	Effect value	SE	95%CI		Indirect effects/direct effects ratio (%)
			LLCI	ULCI	
**Total effect**	−0.170	0.022	−0.214	−0.127	
**Direct effect**	−0.173	0.022	−0.216	−0.130	100
**Ind:emotional regulation capacity-solitude coping-loneliness (suppressing effect)**	0.039	0.011	0.020	0.062	22.54
**Ind:emotional regulation capacity-solitude comfort-loneliness (mediating effect)**	−0.036	0.009	−0.057	−0.020	20.81
**Total indirect effect**	0.003	0.010	−0.016	0.023	1.73

## 5 Discussion

A review of recent analyses of the psychological variable of loneliness reveals a predominant focus from a negative psychology perspective (Twenge et al., 2021; Gniewosz, 2023), often seeking to “eliminate” loneliness through external interventions such as increased social support (Zhang, 2022). This study explores the “dissolution” and “self-reconciliation” of loneliness from an active psychology standpoint, by examining inherent individual psychological strengths (such as emotional regulation and solitude capacity). It aims to address this negative emotion through guidance and acceptance rather than confrontation, potentially offering a new interventional perspective for alleviating loneliness experiences in relevant populations. Firstly, this study indicates differences in emotional regulation capacity among university students based on their gender, grade, place of origin, and only-child status. The solitude capacity of university students was found to be lower than in previous research (Larson and Lee, 1996). In this study, participants scored 40.43 points on loneliness, indicating the presence of relative loneliness among them.

Correlation analysis revealed a negative correlation between emotional regulation capacity and loneliness (Patrichi et al., 2025; Shi and Zhang, 2016). This suggests that stronger emotional regulation ability in adolescents is associated with lower loneliness. Further regression analysis confirmed that emotional regulation ability was negatively associated with loneliness. This aligns with the view that cognitive reappraisal strategies and emotion regulation self-efficacy may be negatively associated with loneliness (Patrichi et al., 2025). Gross's research found that individuals who frequently employ effective emotion regulation experience more positive emotions and fewer negative emotions (McRae, 2020). Students with strong emotional regulation ability may adjust their negative emotions more quickly during solitude, thereby reducing feelings of loneliness. Notably, while the correlation analysis in this study did not find a direct correlation between overall solitude capacity and loneliness, analyzing the sub-dimensions of solitude capacity revealed a positive correlation between solitude coping and loneliness, and a negative correlation between solitude comfort and loneliness. Subsequent regression analyses controlling for potential confounding factors also confirmed that solitude coping significantly and positively correlation with loneliness, while solitude comfort inversely correlation with loneliness. Previous research on the relationship between solitude capacity and loneliness remains debated, with arguments for both positive and negative effects. Some researchers suggest that solitude capacity, by promoting self-reflection and exploration, providing space for personal growth, and fostering a sense of autonomy, leads to more positive emotional experiences and less loneliness (Nikitin et al., 2022; Weinstein et al., 2021; Bradshaw et al., 2025). However, other studies indicate that a greater preference for solitude is associated with stronger loneliness (Sun et al., 2025). Therefore, solitude capacity may function as a double-edged sword, offering clear benefits while also incurring certain costs. Furthermore, different characteristics of solitude capacity have varying effects on loneliness (Toyoshima, 2022). From a lifespan development perspective, the benefits and costs of solitude capacity are dynamic, with the cost of solitude peaking during adolescence. Although excessive solitude time in adolescents is detrimental to establishing and maintaining peer relationships, the benefits of solitude gradually become apparent in early adulthood and solitude capacity is considered a positive developmental characteristic(Coplan et al., 2021). University students are in a transitional stage from youth to adulthood, and the relationship between their solitude capacity and loneliness is also dynamic (Coplan et al., 2021).

Consistent with the above analysis, this study found that solitude coping plays a suppressing role between emotional regulation capacity and loneliness, while solitude comfort plays a mediating role. It should be noted that the direct effect of emotional regulation capacity on loneliness changed only slightly after solitude coping and solitude comfort were included in the model. The total effect was −0.170, whereas the direct effect was −0.173, and the total indirect effect was very small. This pattern suggests that solitude coping and solitude comfort did not substantially alter the overall association between emotional regulation capacity and loneliness at the total-effect level. However, a small net indirect effect does not necessarily mean that solitude capacity is unimportant, because indirect pathways with opposite signs may offset each other in inconsistent mediation or suppression models (MacKinnon et al., 2000, 2007). Specifically, solitude coping showed a positive indirect pathway, whereas solitude comfort showed a negative indirect pathway. These two pathways largely offset each other, resulting in a limited net indirect effect. Therefore, the role of solitude capacity may be better understood at the dimensional level rather than at the total-score level. This finding further supports the need to distinguish solitude coping from solitude comfort when examining the relationship between emotional regulation capacity and loneliness.

This means that while emotional regulation capacity exerts an effect in alleviating loneliness, the concurrently enhanced solitude coping ability partially weakens this protective effect. Conversely, emotional regulation capacity protects against loneliness by enhancing solitude comfort. This may be because individuals with strong emotional regulation capacity can shift their mood more quickly and experience more positive emotions, thereby improving their ability to cope with solitude (Rodriguez and Lumley, 2025). However, strong solitude coping ability may lead individuals to spend more time alone, potentially lacking necessary social interaction (Nikitin et al., 2022). Moreover, being capable of coping with solitude does not necessarily equate to enjoying it. During university years, students face increased social expectations, norms, and peer pressure (Zhao et al., 2025; Gray et al., 2025). In this developmental context, individuals with stronger solitude coping may rely more on being alone to manage stress, which may reduce opportunities for interpersonal interaction and perceived social support. This may help explain why solitude coping was positively associated with loneliness in the present study, reflecting a potential cost of relying on solitude as a coping strategy. On the other hand, individuals with strong emotional regulation capacity may enhance their comfort with solitude through high self-identification (Bradshaw et al., 2025). According to the need-for-solitude theory, individuals internalize solitude as a personal need and view it as a positive state, thereby reducing loneliness during solitude and achieving a positive life state akin to “finding peace by embracing solitude when reaching life's impasses” (Weinstein et al., 2021) (the benefit of enhanced solitude capacity).

The findings of this study suggest that in the process of university students' mental health education and management, the following approaches can be considered: (1) Incorporating theoretical and practical courses on emotional regulation capacity in mental health curricula and focusing on cultivating students' emotion regulation skills; (2) In daily management, paying attention to and guide students with a preference for solitude, especially those with strong solitude coping ability, helping them understand the benefits of solitude capacity while guarding against excessive solitude preference, particularly overly strong solitude coping ability, which may be associated with higher loneliness; (3) Strengthening the creation of collaborative abilities and teamwork environments, providing opportunities for interpersonal interaction, and cultivating students' interpersonal skills to reduce their loneliness.

In summary, solitude coping exerts a suppressing variable between emotional regulation capacity and loneliness, while solitude comfort exerts a mediating **variable**, partially explaining the mechanism through which emotional regulation capacity and loneliness. Future work could focus on cultivating students' emotional regulation capacity to reduce loneliness, as well as developing their solitude capacity to cope with loneliness. However, it is important to emphasize fostering internalized solitude capacity (i.e., solitude comfort) and avoiding excessive externalized solitude capacity (i.e., solitude coping).

This study also has certain limitations. It only examined the relationship between emotional regulation capacity and loneliness. Future research could conduct more in-depth analyses, exploring the mechanisms between different types of emotional regulation capacities and loneliness to ensure systematic and precise investigation. Furthermore, cross-sectional studies cannot conclusively establish causality. Future longitudinal studies could further clarify the mechanism of influence of emotional regulation capacity on loneliness. Additionally, this study sampled from only one university. Although the student population covers multiple provinces and cities, the representativeness of the sample may still be insufficient; furthermore, to the inherent gender structure of the surveyed school's student population, caution is needed when interpreting the conclusions of the difference study based on gender grouping. In the future, the number geographical scope of the surveyed institutions can be expanded to conduct a multicenter study.

## Data Availability

The raw data supporting the conclusions of this article will be made available by the authors, without undue reservation.
